# Supplementary material for: Optimization of High Hydrostatic Pressure Treatments on Soybean Protein Isolate to Improve Its Functionality and Evaluation of Its Application in Yogurt
Source: Foods. 2021 Mar 20;10(3):667. doi: 10.3390/foods10030667 (PMC8003865; doi:10.3390/foods10030667)
Supplement: Supplementary file 1 [file foods-10-00667-s001.pdf]

foods MDPI

Supplementary Materials:

Table S1 showed the coefficients of the models calculated using ANOVA analysis.

Table S1. Analysis of variance (ANOVA) for measured responses of SPI.

Source	Sum of Squares	DF	Mean Square	F Value	*P*-Value (Prob >F)
Analysis of variance (ANOVA) for water holding capacity (WHC)					
Model	4.05	9	0.45	19.79	0.0004 **
X_1	1.40	1	0.084	3.69	0.0960
X_2	0.21	1	1.40	61.66	0.0001 **
X_3	0.084	1	0.21	9.14	0.0193 *
X_1X_2	0.000100	1	0.000100	0.00439	0.9490
X_1X_3	0.021	1	0.021	0.92	0.3684
X_2X_3	0.35	1	0.35	15.56	0.0056 **
X_1^2	0.44	1	0.44	19.49	0.0031 **
X_2^2	0.46	1	0.46	20.09	0.0029 **
X_3^2	0.88	1	0.88	38.65	0.0004 **
Residual	0.16	7	0.023		
Lack of Fit	0.12	3	0.041	4.61	0.0870
Pure Error	0.036	4	0.00893		
Cor. Total	4.21	16			
Analysis of variance (ANOVA) for emulsifying activity index (EAI)					
Model	252.18	9	28.02	11.51	0.0020 **
X_1	82.50	1	82.50	33.88	0.0006 **
X_2	0.20	1	0.20	0.081	0.7835
X_3	2.17	1	2.17	0.89	0.3762
X_1X_2	0.56	1	0.56	0.23	0.6454
X_1X_3	3.12	1	3.12	1.28	0.2953
X_2X_3	3.92	1	3.92	1.61	0.2450
X_1^2	58.76	1	58.76	24.13	0.0017 **
X_2^2	23.82	1	23.82	9.78	0.0167 **
X_3^2	61.14	1	61.14	25.11	0.0015 **
Residual	17.04	7	2.43		
Lack of Fit	12.44	3	4.15	3.60	0.1239
Pure Error	4.61	4	1.15		
Cor. Total	269.22	16			
Analysis of variance (ANOVA) for solubility					
Model	0.080	9	0.00892	20.43	0.0003 **
X_1	0.0000772	1	0.0000772	0.18	0.6867
X_2	0.00178	1	0.00178	4.07	0.0835
X_3	0.014	1	0.014	32.05	0.0008 **
X_1X_2	0.0000210	1	0.0000210	0.048	0.8327
X_1X_3	0.000516	1	0.000516	1.18	0.3131
X_2X_3	0.00440	1	0.00440	10.08	0.0156 **
X_1^2	0.034	1	0.034	78.91	0.0002 **
X_2^2	0.00675	1	0.00675	15.45	0.0057 **
X_3^2	0.013	1	0.013	29.48	0.0010 **
Residual	0.003.06	7	0.000437		
Lack of Fit	0.00203	3	0.000676	2.63	0.1867
Pure Error	0.00103	4	0.000257		
Cor. Total	0.083	16			

* Significant at $p < 0.05$; ** Significant at $p < 0.0001$.
